# Bringing Imaging to the Bedside: The Growing Impact of Point-of-Care Ultrasound in Pediatric and Neonatal Intensive Care

**DOI:** 10.7759/cureus.97874

**Published:** 2025-11-26

**Authors:** Swetha Manoj, Akshitha Talasila, Pallak Khattar, Bhoomika Sastry, Mustafa Samhouri

**Affiliations:** 1 Pediatrics and Neonatology, Government Medical College, Kannur, Pariyaram, IND; 2 Internal Medicine, Mamata Academy of Medical Sciences, Hyderabad, IND; 3 General Practice, Manipal College of Medical Sciences, Pokhara, NPL; 4 Internal Medicine, A.J. Institute of Medical Sciences and Research Centre, Mangalore, IND; 5 Internal Medicine, Ministry of Health (Jordan), Amman, JOR

**Keywords:** cardiology, echocardiography, nicus, picus, pocus, radiology

## Abstract

Point-of-care ultrasound (POCUS) is becoming increasingly integral to pediatric and neonatal intensive care, offering real-time, radiation-free imaging that enhances diagnostic precision and procedural safety. This narrative review synthesizes current evidence on its clinical applications, benefits, training models, and future directions in pediatric intensive care unit (PICU) and neonatal intensive care unit (NICU) practice. POCUS supports rapid hemodynamic assessment, enabling evaluation of ventricular function, preload, afterload, and shock states for timely, targeted management. Lung ultrasound reliably detects pneumothorax, pleural effusion, and consolidations with accuracy comparable to or exceeding chest radiography. Abdominal and renal applications assist in diagnosing trauma, necrotizing enterocolitis, urinary retention, and congenital anomalies, while cranial ultrasound remains essential for identifying intraventricular hemorrhage and hydrocephalus in preterm infants. Procedurally, POCUS improves success and reduces complications during vascular access, thoracentesis, and lumbar puncture. While global adoption is supported by emerging guidelines and competency-based training frameworks, challenges persist, including variability in operator expertise, inconsistent credentialing, limited faculty availability, and gaps in image archiving and quality assurance. Disparities remain between high- and low-resource settings. Advances in handheld devices, artificial intelligence, and tele-ultrasound may expand access and reduce operator dependence, though pediatric validation is limited. Future priorities include multicenter outcome studies, standardized curricula, and cost-effectiveness analyses to guide broader, safe, and equitable implementation.

## Introduction and background

Brief history and evolution of point-of-care ultrasonography (POCUS)

POCUS is a recent development in medicine and is defined as ultrasonography brought to the patient and performed by the provider in real time. Ultrasound technology in medicine has its roots in sound navigation and ranging (SONAR) systems developed during World War I. Over time, this technology was adapted for clinical use, particularly in radiology, cardiology, and obstetrics [1]. Karl Dussik's ultrasound research to see brain structures in the 1940s marked the beginning of clinical adaptation [2]. Wild and Howry then developed A-mode and B-mode scanning, which enabled tissue characterisation and real-time cross-sectional imaging [3,4]. Echocardiography began in the 1950s when Edler and Hertz developed M-mode ultrasonography for cardiac motion measurement [5]. Improvements in pulsed/continuous-wave Doppler and grayscale imaging during the 1960s and 1970s greatly improved cardiac and vascular evaluation [6].

In addition to showing that POCUS could affect treatment choices in about 25% of critically ill patients, the 1993 study by Lichtenstein and Axler marked a turning point because it established a framework for structured lung and critical care protocols and validated the viability, safety, and repeatability of clinician-performed bedside ultrasound [7]. This study demonstrated that ultrasonography might become a dynamic, clinician-operated extension of the physical examination rather than a diagnostic tool exclusive to radiology and cardiology. Additionally, it laid the groundwork for protocolized critical care ultrasonography, which emphasizes quick, problem-focused imaging incorporated into instantaneous decision-making.

Miniaturization significantly changed pediatric and neonatal intensive care as gadgets became smaller. Clinicians were able to confirm vascular catheter tip placement at the bedside, avoid dangerous intrahospital transports, use neonatal-specific probes and fontanelle windows for neuroimaging, and guide real-time changes in ventilation and fluid therapy in critically ill infants thanks to portable and handheld ultrasound [8,9]. The miniaturisation and enhanced portability of real-time ultrasound devices in the late 20th century led to early adoption of POCUS in emergency medicine. It quickly became a preferred tool for managing trauma and shock, offering rapid and potentially more precise bedside evaluations. In the intensive care unit, POCUS was initially used mainly for guiding procedures. As its diagnostic value gained recognition, intensivists began using it to promptly detect critical conditions. 

This qualitative use of ultrasound evolved into standardised protocols like rapid ultrasound in shock (RUSH), focused assessment and sonography in trauma (FAST), and abdominal and cardiac evaluation with sonography in shock (ACES), which streamlined the assessment of shock etiologies. These structured approaches significantly improved timely diagnosis in acute care, paving the way for wider clinical adaptation [10,11]. The acute assessment of trauma and shock was standardized by early structured protocols like FAST, RUSH, and ACES, but their application was mostly adult-focused and constrained by operator dependency, limited specificity, and heterogeneity in picture interpretation, especially outside of prescribed situations [12,13]. 

Distinction between POCUS and conventional radiology-based imaging

Conventionally, ultrasound scans are conducted and interpreted by specialists such as radiologists or cardiologists. In contrast, POCUS is carried out and analysed directly by the treating clinician, enabling more immediate clinical decisions. Its purpose is to serve as a supportive tool rather than a substitute for formal imaging services [14]. POCUS allows clinicians to acquire images almost instantaneously, enabling them to interpret live, real-time scans that align directly with the patient's current symptoms. Unlike traditional imaging workflows, there is no delay for technician-performed scans or specialist interpretation. POCUS can be repeated as needed when the patient's status evolves. It is utilized across numerous medical fields and serves multiple purposes, including guiding procedures, aiding diagnosis, and supporting clinical screening [10,14]

Rationale for its use in pediatric and neonatal intensive care units

The application of POCUS in pediatric intensive care units (PICUs) and neonatal intensive care units (NICUs) is driven by several compelling factors. Children and neonates are particularly vulnerable to the adverse effects of ionizing radiation, making the radiation-free nature of ultrasound an attractive alternative. Additionally, the smaller body size and unique physiology of pediatric patients often allow for superior image quality with ultrasound compared to adults. POCUS enables clinicians to perform serial assessments, monitor disease progression, and guide interventions without the need to transport critically ill patients to radiology suites, thereby minimizing risks associated with patient movement [15-17].

This review aims to synthesise the current literature on POCUS in pediatric and neonatal care, with a particular emphasis on its clinical applications, benefits and limitations, training needs, implementation strategies, and future directions, including technological innovations, research gaps, and the growing potential of POCUS to enhance critical care delivery, particularly in resource-limited settings.

## Review

Clinical application of POCUS in cardiovascular assessment

Echocardiography has emerged as an essential bedside tool for hemodynamic assessment and monitoring in NICU and PICU. It provides real-time information on cardiac function, preload, afterload, fluid responsiveness, pulmonary artery pressure, and cardiac output. As a non-invasive and portable alternative, it offers clinicians the ability to integrate physiological data with clinical findings, allowing for more accurate and targeted therapy [18].

Shock and Hemodynamic Instability

One of the major applications of focused echocardiography in the critical care settings is the assessment of shock and hemodynamic instability. Current expert guidelines highlight the important role of POCUS in determining the underlying cause of shock and distinguishing between distributive, hypovolemic, obstructive, and cardiogenic types, especially in pediatric patients [18,19]. This approach is particularly valuable in neonates and children, where conventional clinical signs of hemodynamic status can often be unreliable [18]. Focused echocardiography allows for rapid bedside assessment of ventricular function, right heart pressures, and pericardial effusion, facilitating accurate classification of shock and timely initiation of appropriate management strategies [19,20]. Persson et al. (2022) found that echocardiography-guided management can significantly influence clinical decision making and improve outcomes in pediatric shock [20]. Neonatologists performing echocardiography offer valuable insights into the underlying mechanisms of circulatory failure and help evaluate the effectiveness of therapeutic interventions, potentially contributing to improved outcomes in NICU [21]. Pediatric data suggest that echo-guided shock management can change therapy and is associated with improved decision-making and timelier interventions; where available, these findings align with reductions in imaging delays and procedure-related complications [18,20].

Volume Status and Fluid Responsiveness

In addition to its role in assessing shock, focused echocardiography is also a valuable tool for evaluating preload and fluid responsiveness in critically ill children. This assessment can be particularly challenging in neonates and mechanically ventilated children, where fluctuating loading conditions and altered lung compliance may affect accuracy [18]. The apical four-chamber view of the heart can be used for qualitative assessment of preload, while quantitative evaluation involves measuring left ventricular volumes and the collapsibility index of the inferior vena cava (IVC) [18]. In spontaneously breathing children, hypovolemia is suggested when the IVC collapsibility index exceeds 50% and the IVC-to-aorta ratio is less than 0.8 [20]. Alternatively, Doppler-based techniques, especially the measurement of velocity time integral (VTI) at the left ventricular outflow tract (LVOT), offer an additional method of quantifying volume status and cardiac output [21]. To measure the VTI, pulse wave Doppler is applied using either the apical five-chamber or apical long-axis view, with the sample volume positioned just below the aortic valve [21]. The VTI reflects stroke distance, which is the distance blood travels during one systolic cycle. When the cross-sectional area (CSA) of the LVOT is calculated using the formula CSA = π × (diameter/2)², the stroke volume (SV) can be determined as SV = VTI × CSA. Cardiac output (CO) is then calculated by multiplying stroke volume by the heart rate (HR) (CO = SV × HR) [12]. Additionally, in ventilated pediatric patients, fluid responsiveness can be reliably predicted by a variation of greater than 15% in the VTI at the LVOT during the respiratory cycles [9]. This approach helps guide fluid therapy more precisely, reducing the risk of both under- and over-resuscitation, which is essential for critically ill children [20].

Advanced Doppler-based assessments (e.g., LVOT VTI trending, RV function indices, and estimation of pulmonary pressures) require training beyond introductory POCUS, including familiarity with neonatal hemodynamics, Doppler angle optimization, and artifact recognition. European Society of Paediatric and Neonatal Intensive Care (ESPNIC) and American Academy of Pediatrics (AAP) guidance recommend performance within structured curricula with image archiving and periodic quality assurance (QA) review, and escalation to pediatric cardiology when a comprehensive evaluation is needed [9,22]. In neonates and ventilated children, IVC and VTI metrics have important caveats. IVC indices are confounded by high positive end-expiratory pressure (PEEP), spontaneous respiratory efforts, elevated intra-abdominal pressure, and right-heart loading conditions; ductal shunting and PFO can further decouple IVC variation from preload. VTI-derived cardiac output is sensitive to LVOT diameter error (squared in the CSA term), suboptimal Doppler angle, and beat-to-beat variability with arrhythmia or ventilator cycling. Accordingly, thresholds should be interpreted with trend-based assessment and integrated with clinical and laboratory markers rather than used in isolation [9,23].

Congenital Heart Defects

Beyond hemodynamic monitoring, focused echocardiography is essential for bedside diagnosis and ongoing management of congenital heart defects (CHD) in the intensive care unit (ICU). It enables rapid detection of anatomical abnormalities such as septal defects, patent ductus arteriosus, and outflow tract obstructions, as well as evaluation of shunt direction and severity, which is crucial for the management of CHD and persistent pulmonary hypertension of the newborn [18,19]. Additionally, routine non-invasive echocardiographic evaluations enable the early detection of postoperative complications, such as pericardial effusion or tamponade, which may require immediate intervention [18,20]. As the clinical focus shifts from cardiovascular to respiratory support, POCUS remains an essential tool for guiding pulmonary assessment and care. While focused cardiac POCUS expedites bedside recognition of ductal physiology and gross structural lesions, it is not a substitute for comprehensive echocardiography in suspected or known CHD. Detailed anatomic delineation, coronary origins, arch anomalies, and pre-operative planning remain within the remit of pediatric cardiology [24].

Pulmonary applications of POCUS

Point-of-care lung ultrasound (LUS) has become a preferred first-line tool for evaluating acute respiratory conditions in neonates and children. It offers a quick, accurate, radiation-free diagnosis at the bedside [20,25,26].

Pneumothorax (PNX)

PNX in pediatric patients can be detected with high accuracy using LUS, which often outperforms chest radiography in both sensitivity and speed [22]. Thoracic POCUS showed high accuracy for clinically significant traumatic pneumothorax, with sensitivity ~87% and specificity 100%, outperforming chest radiography in trauma patients [27]. In a prospective study of children with acute chest pain, the lung point sign demonstrated a sensitivity of 92.3% and a specificity of 100%, while the barcode (stratosphere) sign achieved 100% sensitivity and specificity [28]. Key ultrasound features for diagnosing PNX include the absence of lung sliding, the absence of B-lines, the presence of a lung point, and the barcode sign on M-mode [22,28,29]. In cases of massive PNX, the lung point may not be detectable, highlighting the significance of assessing a combination of sonographic signs for accurate diagnosis [28]. In addition to minimizing radiation exposure and reducing diagnostic delays, LUS allows for timely monitoring of PNX resolution following intervention [29]. These advantages make LUS an invaluable tool in emergency and critical care settings for pediatric patients with suspected PNX [22,29]. Despite these advantages, LUS is inherently operator dependent, and its performance can be reduced in obese patients(attenuation limiting pleural and deep artifact visualization) and in subcutaneous emphysema (air scattering precluding interpretation). Current pediatric guidance recommends structured training, image archiving, and QA to mitigate variability and maintain diagnostic accuracy [9].

Pleural Effusion

LUS offers high sensitivity and specificity for detecting pleural effusions in neonates and children, with diagnostic performance comparable to computed tomography (CT) and superior to chest radiography [25,26]. On LUS, pleural effusions usually appear as anechoic or hypoechoic areas between the visceral and parietal pleura and are best visualized at the dependent costophrenic angle. The diagnosis of pleural effusion is further supported by the presence of the “quad sign” and “sinusoid sign” on M-mode imaging [25]. While signs such as the lung point (PNX) and quad/sinusoid signs (effusion) have high diagnostic value, pediatric inter-observer variability data remain limited, and most reproducibility estimates derive from small cohorts or extrapolation from adult studies-highlighting the need for larger pediatric validation sets [30]. 

Beyond rapid detection and semi-quantification of effusion volume, LUS plays a key role in guiding safe thoracentesis and minimizing procedural complications. Its ability to detect even small effusions and monitor therapeutic response highlights the importance of LUS in the management of critically ill children [26,22]. Compared with CT, LUS shows high accuracy for pleural effusions and pneumothorax and moderate-to-high accuracy for pediatric pneumonia, with meta-analytic estimates supporting LUS as a radiation-free first-line test while CT remains the reference for complex or equivocal cases (e.g., atypical/central pneumonia, interstitial disease, or mixed pathologies) [30,31].

Lung Consolidations

LUS is a reliable bedside tool for detecting lung consolidations, including pneumonia and atelectasis [22,25]. These consolidations appear as tissue-like (hepatized) areas with hyperechoic, branching air bronchograms, which help distinguish them from other causes of respiratory distress [20,25]. Dynamic air bronchograms are usually seen in cases of pneumonia, whereas static air bronchograms suggest atelectasis [20]. Potential pitfalls include false positives when differentiating pneumonia from atelectasis (e.g., impaired ventilation or mucus plugging may yield static air bronchograms and dense subpleural consolidations). LUS patterns must be interpreted with clinical context (fever, leukocytosis, gas exchange) and, when discordant, confirmed with radiography or CT [31]. Focal B-lines may be present in both consolidation and atelectasis, while diffuse B-lines are indicative of pulmonary edema. LUS is as sensitive as chest X-ray for diagnosing pneumonia and offers safe, radiation-free monitoring of disease progression and resolution [20]. In critically ill children, LUS findings should be integrated with the bedside exam and other diagnostics-blood gases, inflammatory markers, chest radiography, ventilator/oxygenation indices-to guide therapy; discordant findings warrant comprehensive imaging to avoid anchoring on a single modality.

Abdominal and renal applications of POCUS

The FAST examination is a key application of POCUS in pediatric critical care, particularly for rapid assessment of free fluid in the peritoneal cavity following blunt abdominal trauma. It is especially useful for repeated evaluations in hemodynamically stable pediatric patients, supporting early detection of intra-abdominal injuries and guiding further management decisions [26]. In children with blunt abdominal trauma, FAST demonstrates variable sensitivity and generally high specificity when compared with CT (reference standard) or formal radiology ultrasound. Pooled pediatric estimates range from sensitivities of 65-89% and specificities of ~97-99%, but single-center cohorts report lower sensitivities in low-volume hemoperitoneum or solid-organ injury without free fluid; thus, a negative FAST does not exclude intra-abdominal injury, and CT or formal ultrasound should be pursued when clinical suspicion persists [32-34].

Necrotizing Enterocolitis (NEC)

POCUS is now widely recognised as a valuable adjunct in the detection and management of NEC in neonates. While definitive diagnosis should involve a radiologist or advanced-trained sonographer, POCUS can reveal critical findings such as bowel wall thickening, pneumatosis intestinalis, portal venous gas, bowel wall perfusion, and free fluid, often outperforming conventional radiography in sensitivity [9]. Despite its benefits, abdominal ultrasonography for NEC is operator-dependent and may overlook mural or modest early perfusion alterations, particularly in preterm infants with less-than-ideal windows. There have been reports of inter-operator variability in the detection of complicated ascites, portal venous gas, and pneumatosis, as well as inconsistent criteria and thresholds among studies that result in inconsistent diagnoses. Standardized scanning procedures and reporting standards are recommended by recent pediatric radiology guidelines in order to enhance repeatability and enable multicenter comparisons [35,36]. According to a meta-analysis, the bowel ultrasound findings that are most strongly associated with poor outcomes in infants with NEC include free air, absent peristalsis, complex ascites, focal fluid collections, bowel wall thinning, and absent perfusion. Early detection of these features can support timely surgical intervention and improved outcomes [35]. Abdominal ultrasound is particularly beneficial when clinical and radiographic findings are inconclusive, as it can identify early or subtle changes and help assess the risk of intestinal perforation [36].

Bladder Volume Estimation

POCUS is a valuable, non-invasive tool for assessing bladder volume in critically ill infants and children. It is especially useful before urethral catheterisation, as it helps determine whether sufficient urine is present in the bladder, thereby reducing unnecessary or failed attempts. Bladder ultrasound can help in assessing urine production and detecting urinary retention in children with oliguria or anuria, providing crucial information for fluid management and further diagnostic evaluation [26,37]. On ultrasound, the bladder typically appears as an anechoic, fluid-filled structure in the anterior pelvis, and even clinicians with minimal training can reliably perform a qualitative assessment [37]. 

Renal Anomalies

POCUS enables rapid bedside evaluation of renal structure and pathology in infants and children. In addition to identifying congenital anomalies such as horseshoe kidney, polycystic kidney disease, and multicystic dysplastic kidney, it is sensitive enough to detect hydronephrosis [38]. Renal ultrasound can also detect urinary tract stones, which appear as echogenic foci with posterior acoustic shadowing, as well as signs of urinary tract obstruction such as pelvicalyceal dilatation and hydroureter [37,38]. In cases of suspected urinary retention or anuria, POCUS can assess post-residual volume and help rule out obstructive uropathy, facilitating timely intervention. While POCUS is extremely helpful for identifying gross renal abnormalities and guiding immediate management, a definitive diagnosis and detailed evaluation often require comprehensive imaging by a pediatric radiologist [9,38].

It can be difficult to distinguish between obstructive and non-obstructive hydronephrosis with POCUS alone; diuretic/duplex regimens and Doppler indices, such as resistive index, improve discrimination but are not always available at the bedside and need expertise. Formal radiological ultrasonography or CT is still required for small (<3-5 mm) or ureteric stones, as well as modest congenital defects (e.g., ectopic ureteral insertion, calyceal diverticula) that may be overlooked on screening POCUS [39-41].

Applications of neurosonography

Intraventricular Haemorrhage

POCUS is the most widely used neuroimaging modality in the NICU and is highly recommended for the diagnosis of germinal matrix haemorrhage and IVH in neonates. It is particularly valuable in extremely preterm infants, where the incidence of IVH is reported to range between 20% and 40% [20,42,43]. Rapid bedside diagnosis of IVH and parenchymal changes using POCUS is essential, particularly in neonates presenting with acute symptoms such as apnea, bradycardia, hypotension, metabolic acidosis, a rapid decline in hematocrit, or seizures. Cranial POCUS is usually performed through the anterior fontanelle; however, adding the mastoid fontanelle window enhances the ability to detect posterior fossa haemorrhages [9,44]. The severity of IVH can be evaluated using established grading systems such as the Papile or Volpe classifications [9,44]. In resource-limited settings, cranial POCUS serves as an especially valuable tool not only for diagnosing IVH but also for guiding decisions regarding the redirection of care [9].

Hydrocephalus

POCUS is also effective for detecting hydrocephalus in neonates by assessing ventricular size and shape [43]. Important sonographic signs that suggest hydrocephalus include widening of the anterior horn of the lateral ventricles (>10 mm), enlargement of the temporal horn (>3 mm), and a third ventricle diameter exceeding 1 mm in the sagittal plane. A bifrontal index of more than 0.5 further supports the diagnosis [43]. Real-time ultrasound is valuable for monitoring the progression of ventricular dilatation following IVH and for evaluating ventriculo-peritoneal (VP) shunt function after placement [44]. Furthermore, transorbital ultrasound measurement of optic nerve sheath diameter provides a non-invasive indicator of elevated intracranial pressure and is useful for monitoring changes following interventions such as VP shunt surgery [45,46]. POCUS can also visualize cerebrospinal fluid flow and detect free fluid in the pelvis, facilitating rapid bedside evaluation in children with suspected shunt malfunction or elevated intracranial pressure [44,47]. In addition to its diagnostic roles, POCUS significantly enhances the safety and success rates of various bedside procedures.

Application of POCUS in procedural guidance

Central Line Placement

POCUS is highly recommended for guiding central venous catheter (CVC) placement and identifying potential complications, particularly in critically ill neonates [42]. Elsayed et al. (2022) reported that chest X-rays may incorrectly identify central line placement in 20-40% of cases, whereas ultrasound can accurately determine whether the catheter tip is intravascular or has migrated extravascularly, without the use of contrast. In cases where central line malposition is suspected to cause pericardial or pleural effusion, POCUS provides rapid and reliable information compared to conventional radiological methods [42]. The use of ultrasound during CVC placement enhances procedural success rates, minimizes complications such as pneumothorax and bleeding, and reduces insertion time. The procedure typically begins with ultrasound mapping of vascular anatomy to identify veins, arteries, and any anatomical variations. Critical steps for safe and effective catheter placement include maintaining continuous visualisation of the needle tip as it enters the target vessel, confirming guidewire placement in both short- and long-axis views, and verifying the final catheter position using ultrasound [42,46,47]. While POCUS-guided CVC placement offers significant advantages, it presents a considerable learning curve for operators. According to Jeon et al. (2024), pediatric surgeons typically need about 15-20 procedures to achieve competency and minimize complications [48]. Davis et al. (2021) highlighted that simulation-based training, which combines theoretical instruction with practical experience, improves procedural skills and safety, both of which are essential for effective clinical adoption [49]. Meta-analyses show ultrasound guidance increases success and reduces attempts and complications for CVC, peripherally inserted central catheter (PICC), and arterial lines in children; competency improves over 15-24 supervised procedures in learning-curve analyses, with measurable drops in time and complications as experience accrues. Operator performance varies by access site and patient size, emphasizing pediatric-specific training [50-52].

Lumbar Puncture

In neonates and children, lumbar puncture is frequently used to diagnose conditions such as meningitis, encephalitis, and intracranial hypertension [37]. POCUS is particularly helpful in neonates with narrow interspinous spaces or in obese children in whom anatomical landmarks are difficult to palpate, as it assists in locating spinous processes, interspinous spaces, and the ideal needle entry point [22,37]. Stewart et al. (2022) conducted a review and meta-analysis comparing ultrasound-assisted lumbar puncture to the traditional landmark technique in infants and neonates. They found that ultrasound guidance resulted in fewer traumatic taps, higher success rates, and shorter procedure times. However, some randomized controlled trials did not report a statistically significant difference in overall success rates [22]. Real-time ultrasound guidance allows clinicians to directly visualize the needle as it advances into the subarachnoid space, further improving procedural safety and precision [22]. Despite these advantages, successful lumbar puncture continues to depend on the operator’s technical expertise. Barsuk et al. (2012) found that simulation-based training significantly enhances lumbar puncture skills, even in residents with minimal prior clinical experience [53]. Their results support the need for standardized assessment of procedural competence before residents are permitted to perform lumbar punctures in clinical practice. Pediatric evidence indicates ultrasound improves first-attempt success in infants, with less consistent benefit in older children; overall success may be unchanged across heterogeneous operators, reflecting inter-operator variability and differing body habitus. Simulation-based mastery learning improves technical performance and reduces traumatic taps, but clinical durability varies without continued practice [54,55]. 

Thoracentesis

POCUS is strongly recommended for guiding thoracentesis in neonates and children, as it improves both the safety and efficacy of the procedure. Ultrasound also reduces the risk of injury to vital structures when inserting a tube or needle by enabling accurate identification of the lung edge, diaphragm, and subdiaphragmatic organs during respiration [42]. In a technical review, Stewart et al. (2022) demonstrated the safety and efficacy of lung ultrasound for guiding chest tube placement or needle aspiration in neonates with pleural effusion or PNX [22]. Ultrasound guidance improves the accuracy of site selection by approximately 26% and significantly reduces the risk of pneumothorax compared to physical examination or chest X-ray [56]. Thoracentesis, though beneficial, has a learning curve that can be overcome with structured simulation-based training. This approach enhances protocol adherence, optimizes ultrasound use, and significantly reduces complication rate. DeBiasi and Puchalski (2016) reported a reduction in pneumothorax rates from 8.6% to 1.1% [57]. Ultrasound guidance increases success and reduces complications (notably pneumothorax) for pleural procedures in children; nonetheless, image quality and needle-tip visualization vary across operators. Thick chest wall, subcutaneous emphysema, and loculations reduce accuracy and may necessitate radiology-performed ultrasound or cross-sectional imaging [58]. 

Benefits and limitations of POCUS in the ICU

Advantages

POCUS is a crucial diagnostic tool. It provides extra details that may aid in reducing the range of possible diagnoses and directing treatment [59]. The emergence of POCUS has significantly advanced the medical profession by allowing non-radiologists to utilize ultrasound at the patient’s bedside [60]. Conventional physical examinations frequently identify a disease only after significant tissue damage has taken place. This is due to the fact that traditional diagnostic indicators were defined when late-stage presentations were prominent. A more sensitive bedside tool like POCUS may facilitate the detection of pathology prior to irreversible organ damage and modify approaches to therapy. For instance, when pericardial effusion is suspected in a hemodialysis patient, acquiring a consultation echocardiography requires time, whereas POCUS provides immediate results and directs treatment. Because conventional physical examinations cannot visualize internal anatomy, they rely on indirect signs. In contrast, POCUS allows for real-time imaging of anatomy and aids in confident clinical decision-making [61].

Accelerated progress in machine learning and artificial intelligence (AI) is beginning to reduce the threshold for fundamental POCUS and sophisticated quantitative assessments. Numerous commercially available POCUS devices provide real-time feedback on image quality. Another advantage is that a novice scanner may produce images of excellent quality with the assistance of AI. Certain POCUS devices now possess the capability to conduct advanced quantitative evaluations autonomously [59]. In critical care situations when formal imaging may not be immediately available, POCUS can prevent unnecessary procedures and treatments, enable more targeted interventions, and deliver prompt diagnostic information. For instance, POCUS can rapidly determine whether a baby’s decreased urine production is the result of oliguria or urinary retention, and this information can help determine whether a urinary catheter or fluid administration would be a suitable solution [62].

In addition to real-time imaging, lung POCUS has numerous advantages over conventional Chest X-ray for diagnosing community-acquired pneumonia, including the elimination of ionising radiation exposure, reduced cost, and enhanced sensitivity and specificity [63]. Given the limited availability of radiologists in emergency situations in intensive care settings, it appears that POCUS-guided suprapubic aspiration (POCUS-SPA) performed by paediatricians is one of the most appropriate and practical diagnostic modalities in newborns with urinary tract infections. A recent study found that POCUS-SPA performed by a physician had the highest success rate in urine sampling in infants compared to suprapubic aspiration and bladder aspiration [60]. This points toward increased procedural safety and higher precision in such settings.

Patients with challenging peripheral venous access sometimes necessitate several attempts and ultimately central venous access, leading to heightened time and resource expenditure. A systematic review and metaanalysis revealed that ultrasound guidance markedly enhanced the success rate and diminished the frequency of skin punctures during peripheral venous access in comparison to conventional approaches [64]. Moreover, the findings indicated that ultrasound guidance does not decrease the duration required for successful cannulation in comparison to traditional approaches. POCUS increases patients’ knowledge of the diagnostic process. It may be well-positioned to improve patients’ comprehension and engagement in the process [65]. POCUS is not designed to substitute consultative imaging; however, it may diminish the necessity for additional diagnostic evaluations in certain patients [61].

Challenges

Despite the demonstrated advantages of a single-location POCUS course, organizing the sessions and optimizing faculty involvement is a logistical difficulty [66]. Over the last three decades, the application of POCUS in critical care medicine has depended on the operator’s level of expertise and training. Because of this, their skill sets can differ greatly. For instance, a patient with hypotension who has a significant decline in left ventricular systolic performance can be diagnosed with cardiogenic shock. To more precisely identify the kind of shock the patient is experiencing, an advanced POCUS user will incorporate a quantitative evaluation of CO as opposed to a basic POCUS user. The scanner’s proficiency with Doppler physics and image acquisition is crucial to measurement accuracy [59].

In pediatric nephrology, ultrasonography guidance for bedside procedures, like dialysis catheter placement, is widely established; however, the application of diagnostic POCUS is still limited [61]. The most significant risk for pediatric specialists doing invasive procedures is probably not knowing how to use an ultrasound during these procedures [67]. During acute therapy, POCUS operators must repeat their patients' assessments and frequently work under time constraints and high stress, particularly in the ICU, emergency department, and operating room. POCUS may result in variability and measurement mistakes. For instance, measuring stroke volume and IVC may be difficult for less skilled POCUS users [68].

If the user does not properly integrate the new information from POCUS into medical decision-making and interpret it in a clinical context, it is irrelevant. Errors in diagnosis and treatment may arise from a lack of focus, poor methodology, incorrect structure identification, or an inability to identify abnormal results [61]. Since ultrasound doesn’t use ionizing radiation to acquire images, it is commonly mentioned as having a desirable safety profile [67]. If not properly maintained, the actual machine might also put individuals at unnecessary risk. Ultrasound probes have the risk of coming into contact with contaminated bodily fluids and spreading harmful pathogens [67,69,70]. Additionally, machine capabilities may be impacted by age and upkeep, like any other piece of equipment. Therefore, even environmental influences on the system may produce less-than-ideal images for procedures and diagnostics [67].

One obstacle to the widespread use of POCUS is worries about missed findings and the ensuing legal consequences [71]. However, studies found no instances of unfavorable legal action against pediatricians, neonatologists, or critical care practitioners for using POCUS for diagnostic purposes [72,73]. On the other hand, lawsuits have been brought about by failing to do POCUS when indicated [73]. Challenges to POCUS adoption for pediatricians include discomfort when performing interpretation of POCUS scans, time constraints, a shortage of machines, and limited access to guidance or quality control from POCUS experts [74]. Additional research in pediatric subspecialties has identified various institutional barriers to the advancement of POCUS, including the implementation of POCUS billing, systems for image archiving, acceptance of non-radiology-performed ultrasound by consultants, and funding for POCUS training [74]. Despite clear benefits, POCUS-guided procedures remain operator dependent; suboptimal probe handling, needle visualization, or misidentification of structures may provide false reassurance in challenging anatomy. Performance is reduced in severe edema, subcutaneous emphysema, and in very small neonatal vessels, where acoustic windows and needle tracking degrade [9].

Quality Control

The shortage of qualified instructors and established protocols from professional organizations continues to hinder the adoption of this expertise and its incorporation into fellowship training programs [61]. Since becoming proficient in POCUS takes time, clinicians should avoid becoming overconfident and promptly seek professional advice when they are uncertain about the sonographic results. This will be made easier with appropriate documentation and image archiving procedures [61]. For POCUS operations to be viable, infrastructure components such as image archiving, documentation, and QA procedures are crucial [67]. In certain sub-specialities, the community lacks standard criteria for its practice, partly because of a lack of knowledge about the enablers and obstacles. Apart from the clinical implementation of POCUS, a variety of useful knowledge regarding the management of training programs, including curriculum design, competency evaluation, and quality assessment, can be acquired by utilising a broad network of professionals both inside and outside one’s own organisation [71].

Like POCUS, practically every aspect of a doctor-patient encounter-such as obtaining a history, performing a physical examination, interpreting test results, and so on-depends on the operator. Therefore, rather than placing the blame on the imaging modalities, emphasis should be placed on effective operator training for POCUS. Developing universal standards for POCUS training would be made easier with formal support and guidance from professional associations [61]. Despite the extensive application of bedside ultrasound in global clinical practice, POCUS in neonatology remains in a nascent stage in many regions of the United States, where neonatologists indicate a restricted availability of equipment and necessary expertise to conduct POCUS [62,75].

Despite the provision of training in numerous institutions, the process remains inadequately structured, and official assessments are rarely conducted [76]. Numerous experts concur that as an increasing number of NICUs acquire ultrasound equipment, it is essential to formulate evidence-based recommendations for utilization, create training requirements, delineate the scope of practice, and implement QA systems [62,76]. Although POCUS lacks a clear definition of QA, it is essential to attempt to provide a continuous learning environment for improving POCUS proficiency [67]. Close cooperation with consultation specialties is essential for safe POCUS, and neonatologists who use ultrasound must have access to training and accreditation systems [75]. Implementing POCUS requires a team effort that includes critical care specialists and other clinical imaging services [62].

Credentialing "defines a physician’s scope of practice and the clinical services he or she may provide and ensures that the physician provides services within the scope of privileges granted” [77]. Physician credentialing for POCUS provides a framework for ensuring appropriate training and integration of ultrasound in clinical practice [53]. Before using POCUS, some programs could require credentialing [62]. Usually granted by the hospital, credentialing can be obtained via practice, education, and training, after which individual physician data is assessed. Providers should be knowledgeable about the POCUS indicators that are relevant to their practice setting, and department administrators should explicitly outline how POCUS will be used in each department [78].

POCUS in critical care settings would benefit from standardized protocols, training guidelines, credentialing, and quality assessment, as noted by our colleagues in fields with established POCUS standards, such as emergency medicine. This would improve program development and establish a mechanism for ensuring optimal use to impact patient care in paediatrics [62]. Paediatricians would be better equipped to recognise POCUS applications that would affect their clinical practice and the possibilities to apply them if they had the necessary tools and training [74].

Training, credentialing, and implementation strategies

Despite global recognition of its value, standardizing POCUS training internationally remains challenging. Major barriers include variability in resources, such as access to ultrasound machines, simulation facilities, and maintenance capacity, as well as regulatory differences governing credentialing, supervision, and billing. Scope-of-practice variations among radiology, cardiology, and intensive care also contribute to inconsistency in curricula and governance models. International bodies such as ESPNIC and the AAP advocate adaptable, competency-based frameworks rather than rigid numeric targets to accommodate diverse resource settings [9]. POCUS has become an indispensable tool in pediatric and neonatal intensive care. In critically ill neonates and children, where other monitoring methods may be limited or invasive, POCUS offers a bedside alternative that enhances diagnostic precision and procedural safety [79]. Recognizing its expanding role, several national and international bodies have begun issuing formal guidelines, training pathways, and implementation models to guide its responsible and standardized use.

Overview of Current Guidelines

ESPNIC issued one of the first international evidence-based guidelines supporting POCUS in neonatal and pediatric intensive care settings. These guidelines recommend using cardiac ultrasound to assess myocardial function and volume status, lung ultrasound for pneumothorax and pleural effusions, cranial ultrasound for IVH and hydrocephalus, and abdominal ultrasound for NEC and free fluid detection. Vascular access and diaphragmatic motion are also addressed. However, ESPNIC emphasizes that POCUS should only be performed by adequately trained providers under a structured framework [9,79].

Similarly, other groups, such as the AAP, have acknowledged POCUS’s potential and outlined that its clinical implementation must include credentialing, documentation, image archiving, and collaboration with radiology and cardiology departments. Despite increasing use in NICU across North America and Europe, standardized credentialing and oversight remain inconsistent, especially in Europe [22,80].

Models for POCUS Training in Residency and Fellowship

POCUS training for pediatric and neonatal care is heterogeneous worldwide. Accredited training programs exist in Canada, Australia, and New Zealand [81]. In the United States, while many providers have acquired POCUS skills and use them in daily practice, gaps in training infrastructure persist. Barriers include a lack of equipment, instructors, protected time, and reimbursement. Institutions in Canada have responded by introducing structured POCUS fellowships in neonatology [81]. Despite the wide adoption of POCUS in Canadian NICUs, only a minority of fellowship programs offer structured teaching. A survey of neonatology program directors and fellows indicated strong interest but highlighted a lack of faculty expertise and time as major barriers [82].

Simulator-based training sessions have shown promising outcomes in improving image acquisition and interpretation accuracy, such as identifying the correct placement of an endotracheal tube (ETT) using a neonatal airway simulator. Studies report significant reductions in interpretation time and error rate with just a few hours of targeted simulation training [83]. Line-tip positioning using POCUS has also demonstrated significant clinical impact. Compared to standard radiography, POCUS improves line accuracy and reduces radiation exposure [84]. These clinical advantages further support structured and widespread training. Targeted echocardiography, particularly in the form of NPE or targeted neonatal echocardiography, has evolved as a crucial bedside tool, enhancing diagnostic accuracy and enabling real-time hemodynamic assessments. Incorporating technology such as AI in this space holds promise for rapid and automated decision-making in critical settings [85].

Integration into Daily ICU Rounds

POCUS, when integrated into daily ICU workflow, enables real-time clinical decision-making and has a significant impact on outcomes. For example, a large academic NICU successfully implemented a structured POCUS program by integrating it into routine care, including vascular access, cranial and cardiac imaging, and daily rounds [86]. Focused cranial POCUS by trained neonatologists enabled improved detection of IVH and other complications in neonates [87]. Such implementation enhanced diagnostic efficiency, with rapid bedside evaluations and reduced delays in treatment. Simulator-based POCUS training has also been shown to help providers identify tracheal vs oesophageal placement of ETTs more accurately, reducing misplacement rates in real-world scenarios [80]. This supports the incorporation of simulator-based modules into residency and fellowship training.

Recognizing the training gap in the United Kingdom, the Children’s Acute Ultrasound (CACTUS) program was recently developed to provide pediatric-specific accredited POCUS education [88]. It highlights the growing need to create subspecialty-specific POCUS training pathways for clinicians working in pediatrics. New clinical protocols, such as the Crashing Neonate Protocol (CNP), demonstrate how POCUS can be used in resuscitative situations to assess pathophysiology and guide interventions when traditional algorithms fail [89]. These protocols are gaining traction as tools for structured and effective POCUS implementation in emergencies.

Quality Assurance and Interdepartmental Collaboration

Establishing robust governance mechanisms is critical to ensuring safe and effective POCUS practice. As of now, no universal credentialing or standard exists for POCUS in neonatology. Therefore, institutions must develop internal systems for risk assessment, oversight, and interdisciplinary collaboration [90]. Risk frameworks must account for the provider’s training, the technology used, and the institutional context. Collaboration between pediatric radiologists, cardiologists, and neonatologists ensures quality review, helps clarify the scope of practice, and fosters educational initiatives [80,90]. Implementing robust QA, image archiving, and documentation processes can be challenging in smaller or resource-limited ICUs. Recommended strategies include centralized image storage on shared Picture Archiving and Communication System (PACS)/Vendor-Neutral Archive (VNA) systems, monthly random audits by credentialed reviewers, integration of structured reporting templates within the electronic health report (EHR), and the use of handheld devices for mobile image capture. Tele-supervision and shared equipment pools can maintain QA continuity when on-site expertise is limited [9].

For successful POCUS integration, a medical director from the Neonatal Division should lead the program, establish its vision, and promote quality through structured metrics such as process and outcome evaluation. Institutional credentialing is essential to ensure provider competency. Until national standards are published, institutions are advised to form hospital-wide POCUS committees that include stakeholders from neonatology, radiology, cardiology, and other relevant departments. Such collaboration ensures that POCUS is used within its defined scope and that interdisciplinary conflicts are avoided through mutual agreement on training requirements and clinical applications. Credentialing, privileging, and defined scopes of practice must be established. POCUS image archiving and documentation are also mandatory to ensure legal protection and facilitate review. QA should include routine review of image quality and interpretation accuracy, with timely feedback to providers [80]. 

Simulation-enhanced curricula have demonstrated improved detection of catheter placement and image interpretation. However, studies indicate that at least 10 ultrasound-guided procedures may be necessary to achieve competency, underscoring the importance of experience and repetition [91]. Affordable and large-scale POCUS programs have been implemented successfully in tertiary NICUs, focusing on improving umbilical venous catheter (UVC) tip identification and central line placements [92]. POCUS can improve outcomes even when used by junior providers. For example, studies have shown that arterial line placements are more successful with ultrasound guidance than with traditional techniques, even when performed by less experienced staff [93]. The evolution of cardiac POCUS in neonatal practice exemplifies how training and governance can enable POCUS to supplement traditional cardiac imaging tools, provided its limitations and scope are clearly defined [94]. Diagnostic POCUS is increasingly viewed as essential for pediatric emergency and critical care. Pediatric practice is moving “beyond the stethoscope” with POCUS offering faster, bedside solutions in dynamic settings [95].

The rapid growth in literature, particularly in pediatric emergency medicine, has cemented POCUS's role as an evidence-based clinical tool for diagnosis and decision-making [37,96]. Lastly, focused cardiac ultrasound has long-standing, evidence-based international recommendations, which should be used to shape current neonatal POCUS strategies [97]. POCUS in neonatal and pediatric care represents a transformative approach to diagnosis and intervention. Its successful integration depends on adherence to international and institutional guidelines, structured training during residency and fellowship, daily integration into ICU rounds, robust QA, and collaboration with radiology, cardiology, and administrative leadership. As simulation, AI, and clinical protocols evolve, POCUS will likely become a core skill for every neonatal and pediatric intensivist, enhancing both bedside care and outcomes in critically ill infants and children [97].

Future directions and research opportunities

POCUS has emerged as a transformative diagnostic and procedural tool in daily practice [98], offering real-time, bedside imaging to support rapid clinical decision-making [99]. With the increasing range of applications for POCUS, there is a rising interest in investigating ways to optimize and standardize it into pediatric critical care practice [100]. Advancements in technology and AI create new possibilities to improve the usability and accessibility of POCUS in low-resource environments [101]. This section explores key areas for future development and research to maximize the impact of POCUS on pediatric critical care.

Emerging Technologies: AI, Handheld Ultrasound Devices, Tele Ultrasound

AI is rapidly transforming the landscape of POCUS by offering powerful tools to assist with image acquisition and interpretation [101]. AI-driven algorithms can assist users in positioning probes, detecting and highlighting anatomical landmarks, and even offering automated measurements and preliminary diagnoses [102-104]. This capability is especially valuable in pediatric critical care, where accurate and timely assessments are essential, yet the skill levels of operators can vary [100]. In resource-limited settings where access to highly trained sonographers or pediatric intensivists may be scarce, AI can serve as a clinical equalizer, enhancing diagnostic capabilities and supporting frontline providers in making critical decisions [101]. However, a significant paradox exists: the low- and middle-income countries that stand to benefit the most from these technological advancements are often the last to receive them [101]. Current AI-enabled features in POCUS, such as probe guidance, image-quality feedback, and automated quantification of ejection fraction, VTI, and B-line counts, have been predominantly trained and validated on adult datasets [105,106]. Validation in neonatal and pediatric critical care settings remains limited, with existing pediatric studies being small-scale and primarily feasibility oriented. For example, AI-assisted ejection fraction estimation has shown good agreement with expert manual measurements in children on extracorporeal membrane oxygenation (ECMO), and pilot studies using AI-augmented lung ultrasound have demonstrated promising diagnostic support, but both were limited by small sample sizes and narrow patient inclusion criteria [107]. Until robust pediatric-specific validation and large-scale datasets are available, these AI outputs should be interpreted as clinical decision-support tools rather than definitive diagnostic measurements [105-107].

Portable, user-friendly handheld ultrasound devices are well-suited for use in resource-constrained or emergency scenarios. Their versatility also enables routine bedside assessments, further enhancing their clinical utility [108]. In addition, tele-ultrasound technology, which enables remote guidance and real-time image review, holds great promise for broadening access to expert support for community hospitals or under-resourced PICUs [109]. These innovations have the potential to democratize access to high-quality ultrasound and improve patient outcomes in pediatric critical care.

Longitudinal Studies on Clinical Outcomes and Cost Effectiveness

While POCUS is now broadly utilized, most research focuses on its immediate diagnostic applications and utility in facilitating medical procedures. POCUS has been integrated into the physical examination of critically ill children within pediatric intensive care units [110]. In adult patients experiencing undifferentiated, non-traumatic hypotension or shock, a POCUS protocol in the emergency ICU led to notable improvements in patient outcomes. Studies indicate that POCUS is associated with reduced hospital discharge times and ICU stays, as well as decreased IV fluid administration, when compared to standard care. Furthermore, the implementation of POCUS has been linked to cost savings through the reduction of CT scan utilization and IV fluid volumes [111].

The structured implementation of POCUS has the potential to optimize resource allocation by diminishing reliance on traditional diagnostic imaging modalities, reducing the duration of mechanical ventilation, and enabling precise management of intravenous fluid administration for critically ill patients within the initial seven days of their intensive care unit stay [112]. Despite its common use, further investigation is needed to determine whether POCUS enhances patient care in the PICU, as current evidence is limited [113]. Evidence gaps include the paucity of large, multicenter pediatric studies validating specific sonographic signs (e.g., lung point, quad/sinusoid, dynamic air bronchograms) against CT or standardized clinical endpoints, and limited data on reproducibility across training levels-priorities for future trials and implementation science [25].

Although adult literature suggests POCUS can reduce CT use, shorten ICU stays, and save costs, there is a paucity of pediatric cost-effectiveness data accounting for device procurement, maintenance, training, and QA. Cost models tailored to pediatric ICUs-especially when factoring neonatal probe sets, simulation training investment, and staff time-are necessary to inform institutional investment decisions [20].

Potential for Expanding POCUS Use in Low-Resource Settings

Medical imaging is a crucial aspect of healthcare; nevertheless, recent research has indicated a substantial lack of imaging resources within low- and middle-income nations [114]. POCUS presents a feasible and scalable approach because it is portable, relatively inexpensive, and simple to operate [115]. Several barriers exist to the adoption of POCUS, including the time investment required to develop technological competence and to incorporate POCUS efficiently into existing clinical workflows [116]. For future initiatives, priority should be given to the creation of PICU implementation models tailored to specific contexts. These models should be backed by research into their clinical impact, provider confidence, and health system outcomes, particularly in settings with limited resources. In resource-limited settings with scarce pediatric cardiology coverage, handheld POCUS, tele-ultrasound, and AI-enabled acquisition support can expand access to time-critical hemodynamic assessment while structured governance and referral pathways mitigate scope-of-practice risks [9]. Adoption may be constrained by capital cost, annual maintenance, and infection-control consumables, with disproportionate impact in smaller or resource-limited ICUs. Shared carts, pooled procurement, basic-to-advanced device tiering, and remote mentorship can improve coverage

Role in Personalized and Precision Critical Care

POCUS has emerged as a valuable tool for delivering precision care [117]. For example, the use of ultrasound-guided evaluation of fluid responsiveness, cardiac function, and pulmonary status can guide the adjustment of fluids, inotropes, and ventilator parameters [111]. This personalized strategy has the potential to improve treatment outcomes for intricate medical situations like pediatric septic shock and acute respiratory distress syndrome. In light of the growing adoption of data-informed approaches within healthcare, subsequent investigations ought to examine the incorporation of POCUS results alongside electronic health records, biomarkers, and artificial intelligence-powered decision-making tools. This integration aims to improve and refine the provision of critical care for pediatric patients [118,119].

## Conclusions

POCUS has become an indispensable extension of bedside assessment in pediatric and neonatal intensive care, enhancing diagnostic accuracy, procedural safety, and clinical decision-making while minimizing radiation exposure and delays in critical interventions. To achieve full integration, future efforts must focus on standardized protocols, international guideline alignment, and measurable evaluation metrics for training and institutional governance to ensure quality and consistency. There remain significant research gaps regarding the long-term impact of POCUS on morbidity, mortality, and resource utilization, warranting well-designed multicenter studies.

Emerging innovations, such as AI-assisted image interpretation, tele-ultrasound connectivity, and handheld devices, hold particular promise for improving access and reliability in cardiovascular, pulmonary, vascular, and procedural applications, especially in resource-limited settings. Ultimately, sustained progress will depend on balancing POCUS use with conventional imaging modalities, fostering collaboration across specialties, and embedding continuous education and quality assurance within critical care systems to ensure comprehensive, safe, and evidence-based patient care.
